# Cytotoxic and apoptotic potential of gemini-chrysophanol nanoparticles against human colorectal cancer HCT-116 cell lines

**DOI:** 10.1186/s40360-022-00597-z

**Published:** 2022-07-23

**Authors:** Alaadin M. Naqishbandi

**Affiliations:** Department of Pharmacognosy, College of Pharmacy, Hawler Medical University, Erbil, Kurdistan Region Iraq

**Keywords:** Chrysophanol, Gemini surfactant, Colorectal cancer, Apoptosis, Bax/Bcl-2

## Abstract

**Background:**

Colorectal cancer is among the most common cancers and accounts for nearly 9% of all cancers in the world. Chrysophanol ﻿is a naturally occurring anthraquinone exerts a number of ﻿pharmacological activities ﻿such as anti-inflammation, anti-cancer, ﻿anti-bacterial, anti-viral, and anti-oxidant effects. ﻿This study aims to ﻿produce a novel gemini chrysophanol nanoparticles (Gemini-Chr NPs), and ﻿to evaluate its anti-cancer effect on the human colorectal cancer cell lines.

**Methods:**

Gemini-Chr NPs were synthesized through nanoprecipitation method and characterized by dynamic light scattering and scanning electron microscopy, ﻿Anti-cancer activities were examined through *MTT* assay on HCT-116 cancer cells, apoptosis was investigated via Annexin V-FITC/PI dual stain assay. Furthermore, the expression of Bax, Bcl-2 and P53 genes were evaluated using real-time PCR and western blotting assay. ﻿

**Results:**

The average particle diameter of the synthesized Gemini-Chr NPs and zeta potential were recorded as 120 nm and 14.4 mV, respectively. In comparison to the normal cells, the cytotoxicity assay confirmed that Gemini-Chr NPs preferentially killed colorectal cancer cells via induction of apoptosis. ﻿Moreover, Gemini-Chr NPs could upregulate the expression of Bax in both cancerous and normal cells (*p* ≤ 0.05) and decreasing the Bcl-2 expression in only tumor cells (*p* ≤ 0.01), while the expression of P53 is modulated in tumor cells (*p* ≤ 0.05).

**Conclusions:**

Gemini surfactants could be considered for efficient delivery and improvement of anti-cancer effect of chrysophanol. Gemini-Chr NPs might have the potential for developing novel therapeutic agent against colorectal cancer.

**Supplementary Information:**

The online version contains supplementary material available at 10.1186/s40360-022-00597-z.

## Background

Colorectal carcinoma considers to be a major cause of deaths due to cancer and accounts for approximately 600,000 death cases per year worldwide [1]. Medicinal plants are widely distributed in Kurdistan region of Iraq that are used traditionally for the treatment of different diseases including cancer [2, 3]. *Rheum* species are known to have medical importance in the treatment of many diseases, ﻿*Rheum ribes* Linn (Polygonaceae) is an Iraqi species of *Rheum* which is also found in Turkey, Iran, Pakistan, Afghanistan, and Russia. Chrysophanol ﻿(1,8-dihydroxy-3-methyl-9,10-anthraquinone), a bioactive molecule in the anthraquinone family which is mainly extracted from *R. ribes* and other species of Rheum [4–7]. Previous studies have reported apoptosis induction of *Rheum* extracts [8, 9], ﻿in addition the root methanol extract of *R. ribes* was recorded with antiproliferative activity on colorectal cancer cell lines, miR-200a/b/c and miR-141 expressions were significant increased (*p* < 0,05) while Bcl-2, ZEB1, and GATA4 expressions were suppressed [10]. Chrysophanol reported to have different pharmacological activities such as anti-inflammatory [11, 12], neuroprotective [13, 14], antibacterial [7, 15]. Furthermore, chrysophanol reported to have activity against breast and colon cancer cells through NF-κB signaling cascades [16, 17], and through AKT and ERK1/2 signaling pathways it induced cell apoptosis in choriocarcinoma [18], also chrysophanol was found to be used as a cancer metastasis inhibitor in the treatment of ﻿colorectal cancer [19]. Product formulations for delivering the naturally derived compounds is important to preserve stability, bioactivity, and bioavailability of the active molecular form, in addition to overcome some other limitations such as preservation and low water solubility, which is the central goal of developing nanoparticles NPs based system. It was reported that chrysophanol NPs significantly minimized tumor size and inhibited tumor growth in BALB/c athymic nude mice. In compared to the free chrysophanol, chrysophanol NPs injection in mice showed higher bioavailability [20]. Gemini surfactants are consisting of two identical conventional surfactants that are linked together by a flexible or rigid spacer, and found to be very effective as drugs delivering system into cells [21]. Therefore, as a suitable way to improve chrysophanol anticancer activity, this research aims to explore the effects of gemini surfactant-chrysophanol (Gemini-Chr) against human colorectal cancer HCT-116 cell lines and to study the possible molecular mechanisms associated with apoptosis induction.

## Materials and methods

### NPs preparation and characterization

**Fig. 1:**
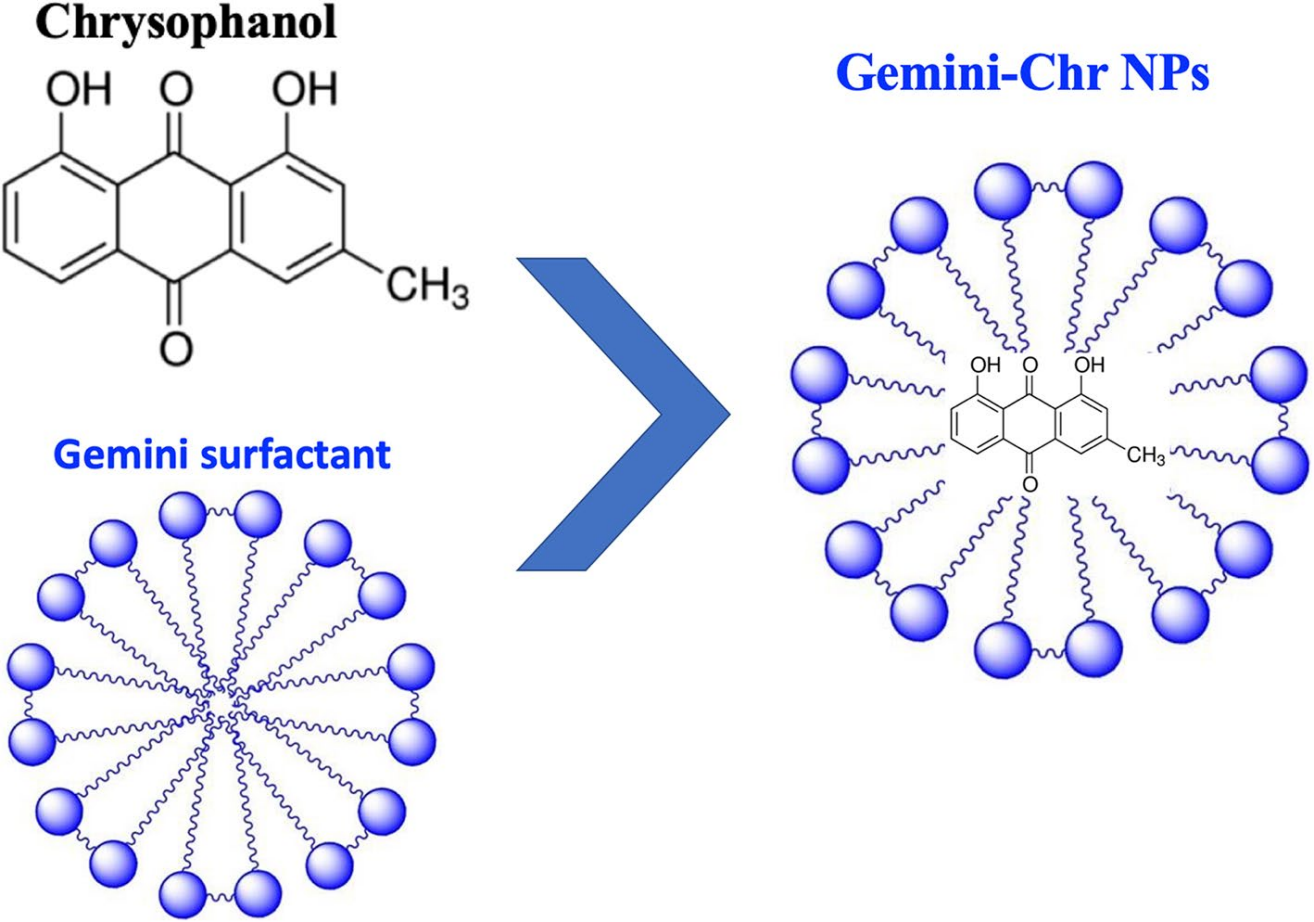
The structural representation of a novel Gemini-Chr NPs

The preparation of Gemini-Chr NPs was carried out by nanoprecipitation method [22]. Briefly, 5 mg of chrysophanol (CAS no.: 481–74-3, Sigma Aldrich, USA) and 100 mg of methoxy-poly (ethylene glycol) urethane Gemini surfactant were dissolved in 5 mL methanol (Merck). After evaporation of methanol by rotatory evaporator, the solution is filtered by 0.2 μm syringe filter, lyophilized, and stored at 4 °C until further use. Chrysophanol was dissolved in phosphate-buffered saline (PBS) before each treatment. Dynamic light scattering (DLS), which is applied for the physicochemical characterization of nanoparticles, was used to measure the zeta potential as well as the hydrodynamic diameter of the Gemini-Chr NPs. Additionally, scanning electron microscopy (SEM) achieved detailed visual image of the Gemini-Chr NPs with high-quality and spatial resolution for characterization and a better understanding of cytotoxicity mechanism [23]. The structural representation of a novel Gemini-Chr NPs is shown in Fig. 1.

### Cell culture

In this study, human colorectal cancer cell lines (HCT-116) and Mouse Embryonic Fibroblast normal cells (MEF) were obtained from the National Cell Bank of Iran (Pasteur Institute, Tehran, Iran). DMEM (DMEM/HG; Gibco) with high glucose content culture medium was used to expand the cells. Fetal bovine serum 10% (FBS, Gibco, USA) and Pen-Strep solution 1% (Biochrom GbmH, Berlin, Germany) were added to the basal medium. Cells between passages 3–6 were used for different analyses.

### Cell viability assay

The viability of HCT-116 and MEF cells were evaluated after treatment with Gemini surfactant NPs, Chrysophanol, and Gemini-Chr NPs using *MTT* (3-(4, 5-dimethylthiazole-2-yl)-2, 5- diphenyltetrazolium bromide) method [24]. In brief, an initial number of 1 × 10^4^ cells were suspended in 100 mL of DMEM/HG in each well of 96-well plates containing 10% FBS. Cells were incubated with different ﻿concentrations of Gemini surfactant NPs, Chrysophanol, and Gemini-Chr NPs for 24, 48 and 72 h. Supernatants were discarded and replaced with of *MTT* solution 5 mg/mL. Cells were kept for 3–4 h at 37 ˚C and Dimethylsufoxide (DMSO) solution was added. A microplate reader (Bio-rad, England) was used to read the optical density of each group and expressed as % of the control group.

### Flow cytometry analysis

Annexin V-FITC/PI double staining assay was applied to determine apoptosis, using an Annexin V-FITC/PI apoptosis detection kit (BD Pharmingen, San Diego, CA, USA). Gemini-Chr NPs 40 and 60 µM were added to colorectal cancer cells HCT-116 and cultured for 24 h, collected and resuspended in 0.5 mL binding buffer/sample for 5 min. Then 5 μL each of FITC-labeled Annexin-V and PI were added for 15 min at 37 °C in dark. Flow cytometer (Becton Dickinson FACS, Holdrege, NE (Nebraska), USA) was used to analyze the stained samples using FlowJo 7.6.1 Software.

### Gene expression analysis

To perform reverse transcription polymerase chain reaction (RT-PCR), total RNA was extracted from both of the control and treated cells using TRIzol™ reagent (Bioneer, Korea) according to the manufacturer’s guideline, picodrop spectrophotometer (Thermo) and 1% agarose gel electrophoresis were used to determine the quality and concentration of RNAs. Easy™cDNA Synthesis Kit (Takara Co., Japan) was used to synthesize complementary DNA (cDNA) by the oligo-dT method following the manufacturer’s instructions. The genes expressions of Bax, Bcl-2 and p53 were done using SYBR Green (Ampliqon, Denmark) RT-PCR analysis using appropriate primers. The primers were designed using Oligo7 software (Table 1). In this study, the total volume for RT-PCR reaction reached 10 μL consisted of 5 μL of SYBR Green PCR master mix, 1 μL of forward and reverse primers, 1 μL of cDNA template, and 3 μL of double distilled water (ddH2O) [24]. Relative gene expression was normalized to β2m as an internal control, and calculated by using the 2^−ΔΔCT^ method [25].

**Table 1:** Sequences of primer pairs used in PCR

Gene	Sequence (5′ → 3′)	Amplicon (bp)
Bax	Forward: 5' GCAAACTGGTGCTCAAGG 3' Reverse: 5' ACTCCCGCCACAAAGA 3'	187
Bcl-2	Forward: 5' TGGGAAGTTTCAAATCAGC 3' Reverse: 5' GCATTCTTGGACGAGGG 3'	236
P53	Forward:5'CACCTACCTCACAGAGTGCAT 3' Reverse:5'AAACTACCAACCCACCGACCA 3'	146
β2m	Forward: 5' CTACTCTCTCTTTCTGGCCTG 3' Reverse: 5' GACAAGTCTGAATGCTCCAC 3	191

*bp* Base pair

### Western blotting

HCT-116 and MEF cell lines were treated with 40 and 60 µM of Gemini-Chr NPs, lysed using 500 μL lysis buffer. Then from each group, 10 μg protein was electrophoresed using 10% SDS-PAGE at 120 V for 45 min and then transferred on to membranes of polyvinylidene difluoride at 120 V for 1.5 h and incubated with Bax (Cat no: sc-7480; Santa Cruz Biotechnology, Inc.), Bcl-2 (Cat no: sc-492; Santa Cruz Biotechnology, Inc.) and p53 (Cat no: sc-126; Santa Cruz Biotechnology, Inc.), at 4 °C for 24 h. The membranes after PBS wash for 3 times were incubated for 1 h at room temperature with appropriate HRP-conjugated secondary antibodies (Cat no: sc-516102 and sc-2357; Santa Cruz Biotechnology, Inc.). The detection of immunoblots were carried out on X-ray films using chemiluminescence ECL solution (Bio-Rad). The relative protein expression was normalized to β-actin (Cat no: sc-47778; Santa Cruz Biotechnology, Inc.) [24].

### Statistical analysis

All values represent the mean ± standard deviation of three independent experiments. The one-way analysis of variance (SPSS software v.22.0) was employed to analyze data statistically. Groups considered statistically significant when *P* < 0.05. GraphPad PRISM version 6.01 software was used for generation of graphs.

## Results

### Gemini-Chr NPs physicochemical characteristics

**Fig. 2:**
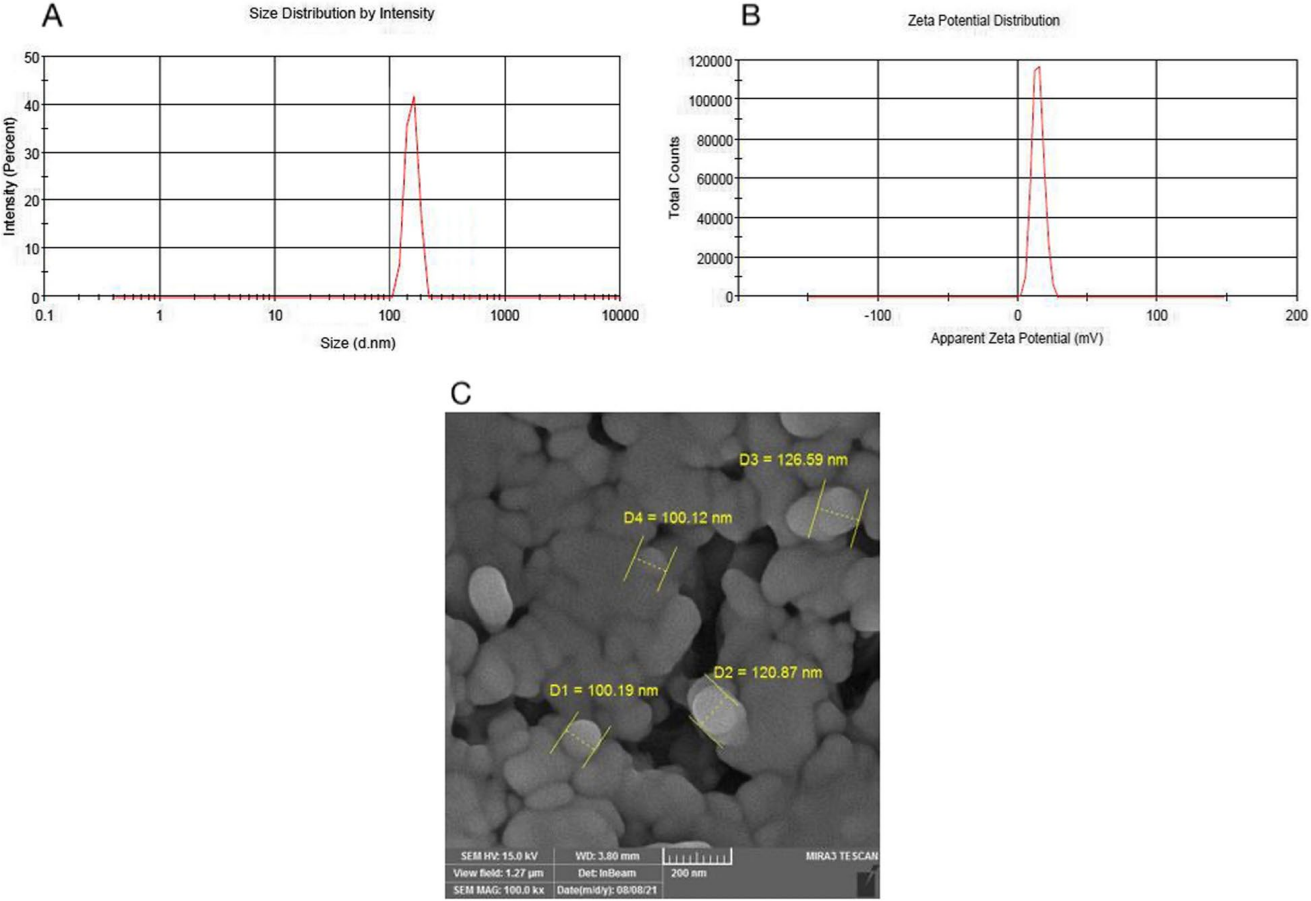
Physical characterization of Gemini-Chr NPs. **A** Cluster size of Gemini–Chr NPs obtained from DLS measurements. **B** The zeta potential of Gemini–Chr NPs. **C** SEM imaging was used to measure mean size of nanoparticles

Gemini-Chr NPs synthesis and characterization were carried out as described previously, the cluster size distribution obtained from DLS analysis (Fig. 2A) shows the average hydrodynamic diameter to be 120 nm. In addition, zeta potential of Gemini-Chr NPs was calculated to be 14.4 mV which is accepted value for stable dispersion (Fig. 2B). SEM micrograph showed that Gemini-Chr NPs were well separated from each other and spherical in shape (Fig. 2C).

### Gemini-Chr NPs cellular toxicity

**Fig. 3:**
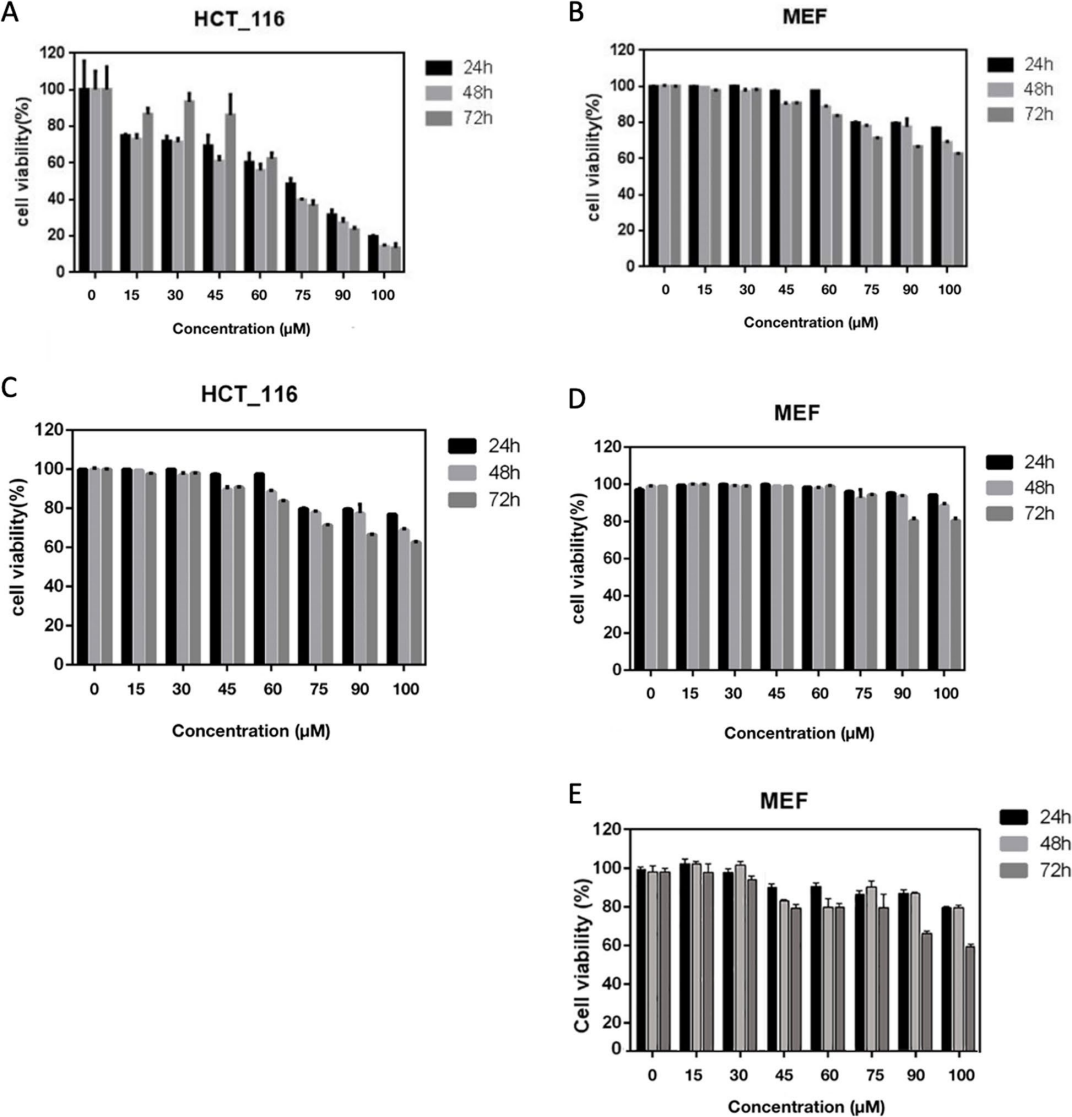
The growth-inhibitory effect of Gemini-Chr NPs (**A** and **B**), Chrysophanol (**C** and **D**), and Gemini surfactant NPs (**E**) on HCT-116 and MEF cell lines using MTT-based assay. Cells were treated with Gemini-Chr NPs, chrysophanol, and Gemini surfactant NPs in a time- and dose-dependent manner. Data represent mean ± standard deviation of three independent experiments

*MTT* assay showed that Gemini–Chr NPs inhibit the proliferation of HCT-116 cancer cell lines in a dose and time dependent manner, (Fig. 3 A). The IC_50_ of Gemini-Chr NPs on 24, 48, and 72 h in HCT-116 cells was recorded as 60.17, 58.52, and 60.80 µM (*p* < 0.05), respectively. Furthermore, viability assay showed that Gemini-Chr NPs does not affect normal MEF cells in the IC_50_ values of cancerous cells. The IC_50_ values for MEF cells were recorded for concentrations more than 100 µM (Fig. 3 B). Free chrysophanol did not show any toxicity on both HCT-116 and MEFs cells in the concentrations that have been employed for Gemini-Chr NPs (Fig. 3 C and D) and the same thing was recorded for Gemini surfactant NPs on MEFs cells (Fig. 3 E).

### Apoptosis assay by flow cytometry

**Fig. 4:**
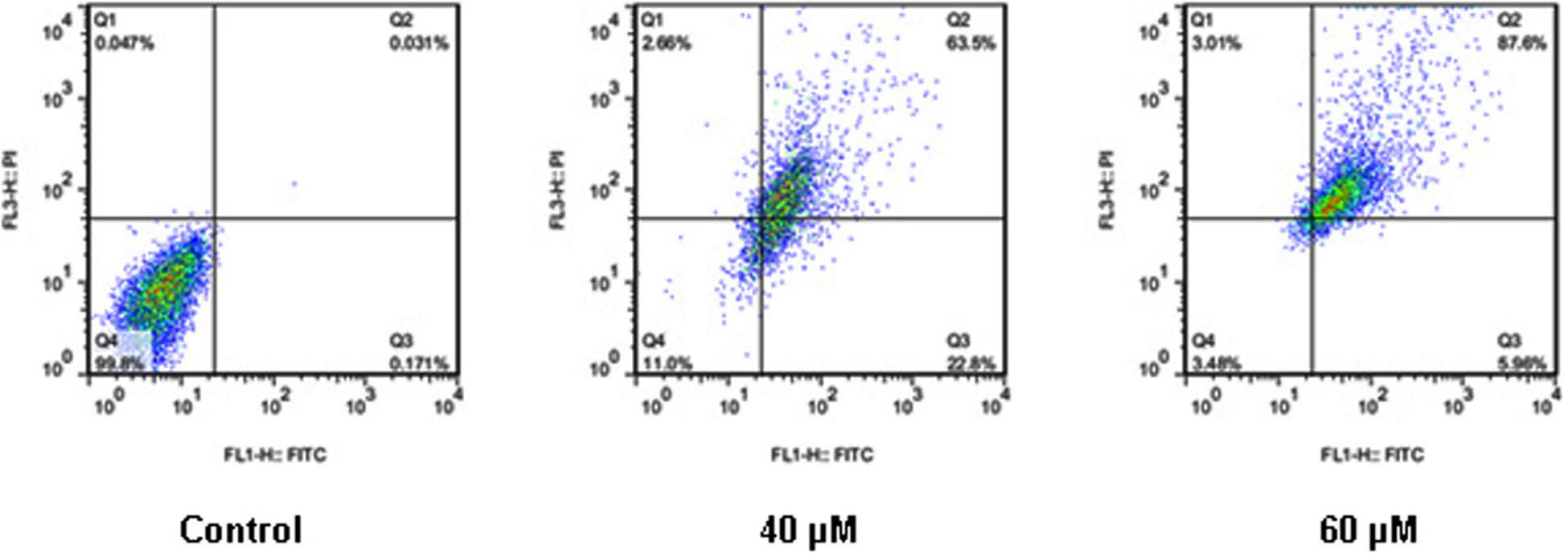
Flow cytometry analysis of mode of cell death in Gemini-Chr NPs treated colorectal cancer cells. Annexin V-FITC/propidium iodide (PI) histograms were obtained from analysis of colorectal cancer HCT-116 cells incubated with 40 and 60 µM of Gemini-Chr NPs for 24 h

Annexin V/FITC assay was employed to study the mode of death in Gemini-Chr NPs treated cells. The results of annexin V-FITC positive cells indicated significantly increased apoptosis of Gemini-Chr NPs treated cells (Fig. 4).

### Bax, Bcl-2, and P53 genes expression

**Fig. 5:**
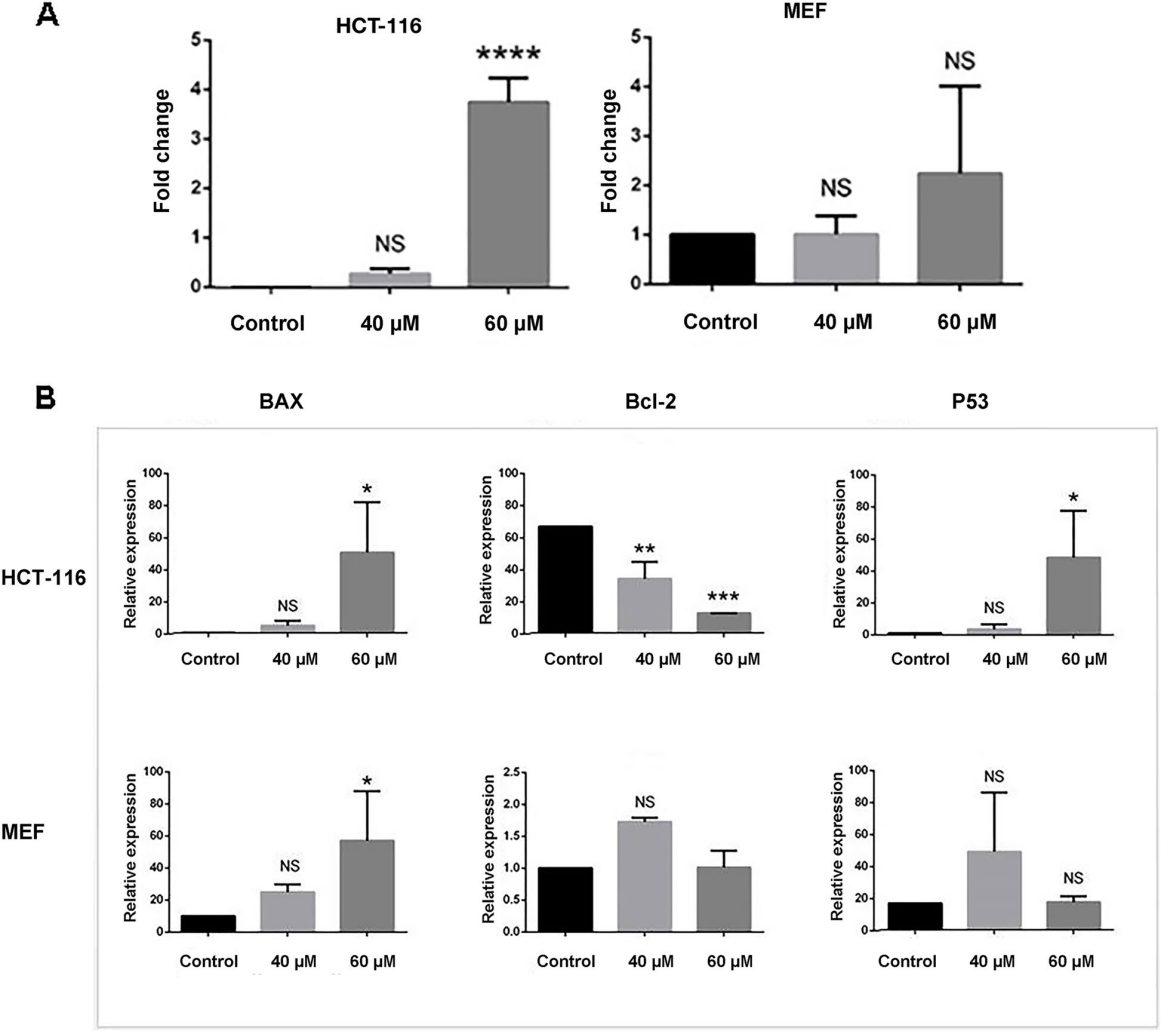
**A** Expression ratio of Bax/Bcl-2 in non-tumoral MEF and HCT-116 tumor cells. ****: *p* value ≤ 0.0001; B: Relative expression of Bax, Bcl-2 and P53 as apoptotic genes in both cell lines. *: *p* value ≤ 0.05, **: *p* value ≤ 0.01, ***: *p* value ≤ 0.001

Real-time PCR was used to further confirm the mode of cell death through the study of apoptotic genes expression, it was revealed that Bax/Bcl-2 expression ratio, a hallmark of apoptosis, was elevated in a dose-dependent manner (Fig. 5A, *p* ≤ 0.0001). As Fig. 5B illustrates, Bax is over-expressed in both cancerous and normal cells (*p* ≤ 0.05). However, Bcl-2 as an anti-apoptotic gene is down-regulated in only tumor cells (*p* ≤ 0.01). Furthermore, the analysis demonstrated that the expression of P53 is significantly modulated in tumor cells rather than normal ones (*p* ≤ 0.05).

### Bax, Bcl-2, and P53 proteins expression

**Fig. 6:**
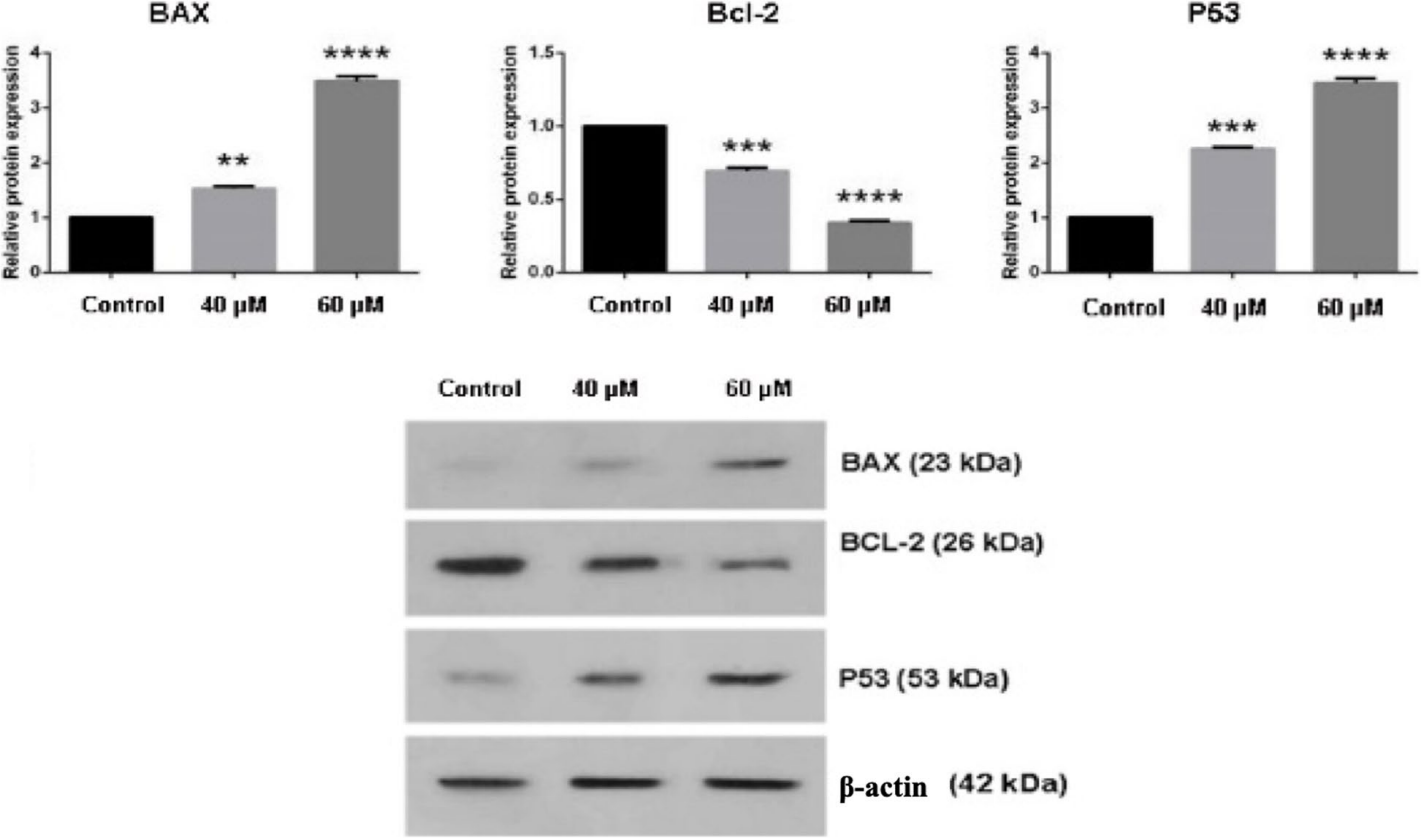
The expression of Bax, Bcl-2, and P53 in HCT-116 cells treated with 40 and 60 µM of Gemini-Chr NPs for 24 h detected by Western blot analyses. The bands show the expression of proteins in tumor cells. The relative protein expression was normalized to β-actin. Full-length blots/gels are presented in supplementary figure S1. **: *p* value ≤ 0.01, ***: *p* value ≤ 0.001, ****: *p* value ≤ 0.0001

Given that apoptosis inducing agents frequently signal through changes in the expression of Bcl-2 and non Bcl-2 related proteins. Thus, expression of anti-apoptotic Bcl-2 and pro-apoptotic Bax and P53 proteins before and after treatment with Gemini-Chr NPs at 40 and 60 µM was examined in HCT-116 cell lines using western blot analysis (Fig. 6). The results revealed reduction in Bcl-2 expression (*p* value ≤ 0.0001) while increased expression of Bax and P53 in dose dependent manner (*p* value ≤ 0.0001).

## Discussion

Colorectal cancer is the third most diagnosed malignancy with high unacceptable mortality, current chemotherapy drugs which although effective but induce severe side effects to the patients, therefore novel approaches for the treatment of colorectal cancer are needed [26]. The use of plant-based drugs has exhibited incomparable advantages in various fields due to their unique chemical structures and diverse biological and clinical applications [27]. Chrysophanol was reported in several cancer cells to inhibit cell growth and regulate genes and proteins involved in controlling apoptosis, cell invasion, metastasis and cell cycle arrest [28]. The application of NPs as carriers or delivery systems has enhanced the conventional anticancer treatments due to their specificity to cancer cells, that enhance drug efficiency and decrease systemic toxicity [29, 30]. In this study, Gemini-Chr NPs were successfully produced and ﻿the results revealed cytotoxicity and cell apoptosis induction in HCT-116 cells. Previous records showed no cytotoxicity of Gemini surfactants on HCT-116 cells [31], and the results of this study showed that Gemini surfactant is a little bit toxic than Gemini-Chr NPs against MEFs cells but non significant and this is probably due to the smaller size for Gemini surfactant that can more easily enter the cells. Gemini surfactant due to its biocompatibility and biodegradability properties can be used for administrating hydrophobic drugs as a safe and cost-efficient system [23], and in comparison with monovalent surfactants offer some advantages such as low critical micellization concentration and high solubilization power [32]. The physicochemical characteristics of NPs are essential for their cellular uptake, and moreover the size and charge of NPs are important properties that could affect their pharmacological properties [33]. Studies showed that NPs with 10–250 nm diameter are convenient for systemic administration due to increase in permeability and overcome physiological barriers [34]. The synthesized Gemini-Chr NPs with 120 nm diameter sphere shaped and 14.4 mV zeta potential giving the possibility to be used for systemic circulation applications with proper dispersion without accumulation, appropriate dimension, and surface charge. The results of *MTT* assay showed that Gemini-Chr NPs decreases the cell viability of HCT-116 cancer cell lines both in a dose and time dependent, while free chrysophanol did not show a considerable inhibitory effect [35, 36]. Therefore, it was supposed that the activity of chrysophanol is increased significantly which may be due to enhancement of its solubility, uptake, and cytotoxicity using gemini surfactant nanocarriers, further future experiments should be carried out to support this result. Furthermore, Gemini-Chr NPs showed no significant difference in normal MEF cells, indicating its low toxicity. Apoptosis, a programmed cell death, is a tightly regulated process that does not affect bordering cells and is considered as a good target for anticancer treatment [37, 38]. The researchers investigated previously the role of chrysophanol in the modulation of various apoptosis pathways in cancer cells [16, 18, 39]. Flow cytometry analysis revealed that Gemini-Chr NPs induced apoptosis in HCT-116 cells using Annexin V/FITC assay. These data at least show that, similar to previous reports, Gemini-Chr NPs through different pathways induce apoptosis. The Bax/Bcl-2 ratio is considered a critical factor in apoptosis. RT-PCR and western blotting were used to investigate the expression of P53 and its related genes Bcl-2 and Bax before and after treating with Gemini-Chr NPs. The ratio of Bax/Bcl-2 expression is the determining factor for the induction of apoptosis, the results showed that the ratio of Bax/Bcl-2 expression was significantly increased (*p* ≤ 0.0001) dose-dependently after addition of Gemini-Chr NPs, which confirmed the susceptibility of HCT-116 cells against apoptosis [20]. Furthermore, the analysis demonstrated that the expression of P53 is significantly modulated in HCT-116 tumor cells rather than normal ones. Through control of cell proliferation and apoptosis, the tumor protein P53 has an essential role in tumor development. It was reported that chrysophanol inhibited the proliferation of human mast cells by enhancing P53 protein level [40],in the same line chrysophanol by promoting reactive oxygen species (ROS) has efficacy to induce apoptosis in P53-expressing cancer cells [41]. It was found that increased P53 activity and ROS production occurred in prostate cancer LNCap cells treated with chrysophanol NPs which relies generally on dramatic alterations in mitochondrial morphology during the early stages of apoptotic cell death that involve the network fragmentation and the remodeling of cristae [20]. The increasing in P53 gene activity will activate P21 transcription factors and this prompts binding of cyclin dependent kinase 2 (CDK2) with cyclin E leading to cell cycle stop. Also, the activation of P53 in the cytosol will activate Bax and cause suppression of Bcl-2, and leads to change in permeability of mitochondrial membrane and causes cytochrome c to exit to the cytosol. The reaction of cytochrome c with protease activating factor-1 (APAF-1) through activation of cascade and caspase reaction will triggers DNA-se activation. The DNA-se in turn enters the nucleus leading to fragmentation of the DNA, cleavages of poly ADP-ribose polymerase (PARP), and apoptosis [42]. This research is the first study on evaluation of Gemini-Chr NPs effects against HCT-116 cells growth and apoptosis. However, further molecular analysis needs to be performed to confirm how Gemini-Chr NPs may affect the protein pathways.

## Conclusion

Gemini-Chr NPs were successfully synthesized and the cytotoxicity assay suggested their preferential proliferation inhibition and reduced cell viability of HCT-116 colorectal cancer cells in comparison to the normal cells ﻿via induction of apoptosis. Additionally, Gemini-Chr NPs upregulated the expression of Bax and p53 but downregulated Bcl-2 expression in HCT-116 cells. Hence, Gemini-Chr NPs might have the potential for developing novel therapeutic agent against colorectal cancer.

## Supplementary Information

**Additional file 1: Figure S1.** The uncropped full-length gels and blots for Fig. 6 in HCT-116 cells. Each lane was labelled according to the cropped gels/blots in Fig. 6.

## Data Availability

The data that support the findings of this study are available on request from the corresponding author. The data are not publicly available due to privacy or ethical restrictions.

## Abbreviations

APAF-1: Apoptotic protease activating factor-1
CDK2: Cyclin dependent kinase 2
cDNA: Complementary DNA
ddH2O: DDouble distilled water
DLS: Dynamic light scattering
DMSO: Dimethylsufoxide
Gemini-Chr NPs: Gemini chrysophanol nanoparticles
HCT-116: Human colorectal carcinoma cell lines
MEF: Mouse embryonic fibroblast normal cells
MTT: 3-(4, 5-Dimethylthiazole-2-yl)-2, 5- diphenyltetrazolium bromide
PARP: Poly ADP-ribose polymerase
PBS: Phosphate-buffered saline
PCR: Polymerase chain reaction
SEM: Scanning electron microscopy
