# Supplementary figures and images for: Cytotoxic and apoptotic potential of gemini-chrysophanol nanoparticles against human colorectal cancer HCT-116 cell lines
Source: BMC Pharmacol Toxicol. 2022 Jul 23;23:56. doi: 10.1186/s40360-022-00597-z (PMC9308237; doi:10.1186/s40360-022-00597-z)

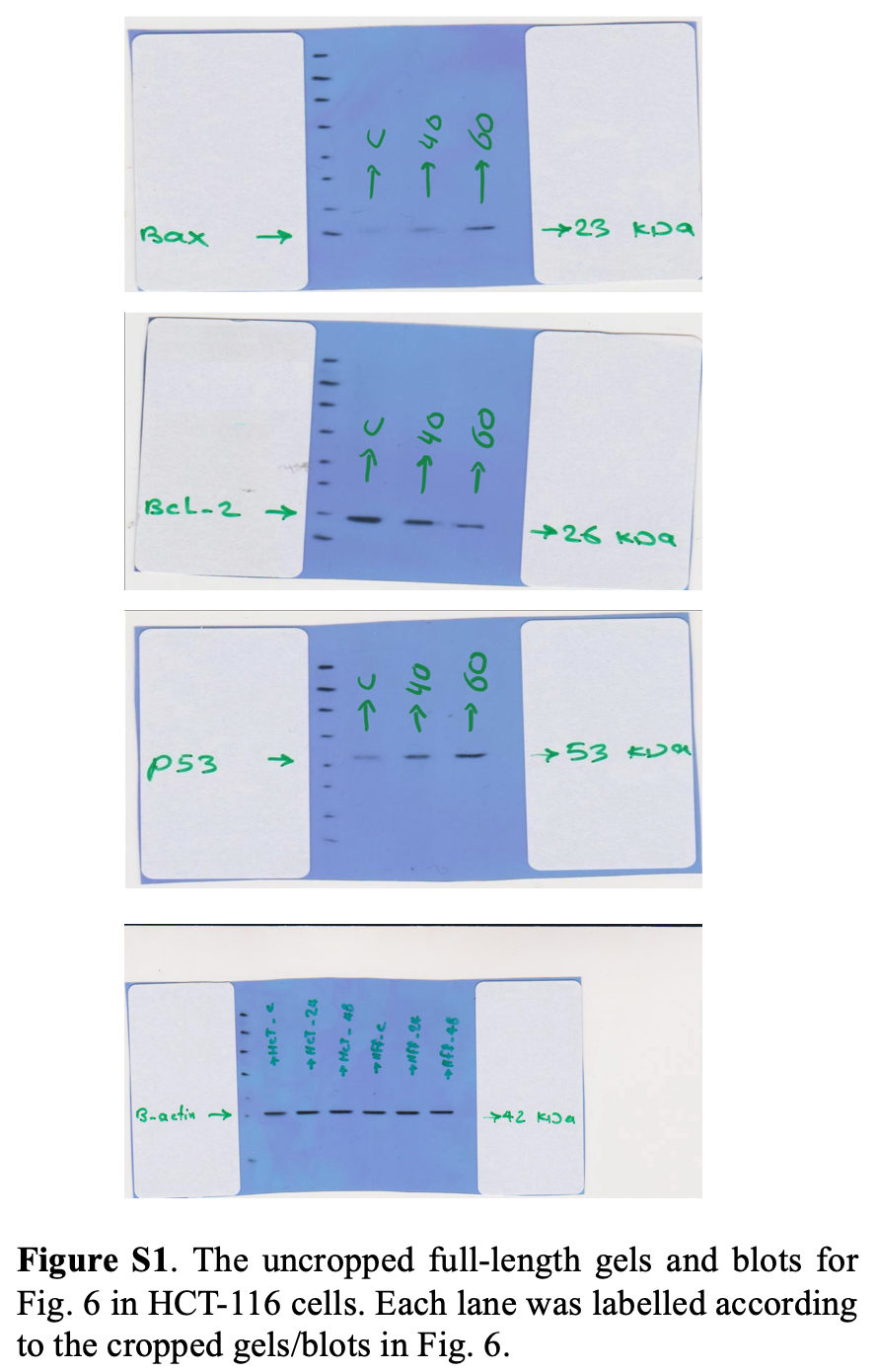

Supplement: Supplementary file 1 — Additional file 1: Figure S1. The uncropped full-length gels and blots for Fig. 6 in HCT-116 cells. Each lane was labelled according to the cropped gels/blots in Fig. 6. [file 40360_2022_597_MOESM1_ESM.jpg]
